# N-Myc-induced up-regulation of TRPM6/TRPM7 channels promotes neuroblastoma cell proliferation

**DOI:** 10.18632/oncotarget.2283

**Published:** 2014-07-31

**Authors:** Zheng Zhang, Malika Faouzi, Junhao Huang, Dirk Geerts, Haijie Yu, Andrea Fleig, Reinhold Penner

**Affiliations:** ^1^ Center for Biomedical Research, The Queen's Medical Center, University of Hawaii Cancer Center and John A. Burns School of Medicine, University of Hawaii, Honolulu, U.S.A; ^2^ Department of Pediatric Oncology/Hematology, Erasmus University Medical Center, Dr. Molewaterplein 50, GE Rotterdam, the Netherlands; ^3^ Department of Pharmacology, School of Pharmaceutical Sciences, Central South University, Changsha, Hunan, China

**Keywords:** cancer, magnesium, neuroblastoma, N-Myc, TRPM6, TRPM7

## Abstract

Intracellular levels of the divalent cations Ca^2+^ and Mg^2+^ are important regulators of cell cycle and proliferation. However, the precise mechanisms by which they are regulated in cancer remain incompletely understood. The channel kinases TRPM6 and TRPM7 are gatekeepers of human Ca^2+^/Mg^2+^ metabolism. Here, we investigated the human neuroblastoma cell line SHEP-21N in which the MYCN oncogene (encoding N-Myc) can be reversibly expressed under control of an inducible repressor. We report that N-Myc expression increases cell growth and up-regulates both TRPM6 and TRPM7 expression. Membrane current analyses reveal that endogenous TRPM6/TRPM7 currents exhibit reduced Mg·ATP suppression, increased Mg^2+^ sensitivity, and diminished sensitivity to 2-APB inhibition. These properties are consistent with N-Myc-induced increase of heteromeric TRPM7/TRPM6 channels promoting Ca^2+^ and Mg^2+^ uptake. Genetic suppression of TRPM6/TRPM7 through siRNA inhibits cell proliferation, suggesting that N-Myc can promote neuroblastoma cell proliferation through up-regulation of divalent cation-transporting channels.

## INTRODUCTION

Both Ca^2+^ and Mg^2+^ are critically involved in essentially every single step of cell proliferation, with cancerous cell growth representing a harmful form of deregulated proliferation. Interestingly, cancerous tissue acts as a Mg^2+^ trap at the expense of plasma or surrounding tissues, suggesting significant uptake by cancer cells [1-3]. The precise mechanisms by which divalent cations regulate cell proliferation remain to be elucidated, but recent evidence indicates critical involvement of the Ca^2+^- and Mg^2+^-transporting transient receptor potential melastatin-related ion channels TRPM7 and TRPM6 [4-6]. TRPM7 and TRPM6 are channel kinases possessing both a Ca^2+^/Mg^2+^-permeable ion channel pore and a carboxyl-terminal atypical α-kinase [4,7,8]. The two channels are highly analogous in many aspects, such as channel selectivity [9,10], current-voltage relationship, as well as modulation by acidic pH [7,9,10], extracellular divalent cations [9,10], and PIP_2_ [11,12]. However, recently, they were shown to be differentially regulated by the channel blocker waixenicin A [13], 2-aminoethoxydiphenyl borate (2-APB) [9,14], intracellular halides [15], as well as intracellular Mg^2+^ and ATP [14].

While TRPM7 is ubiquitously expressed and the native TRPM7-like current MagNuM (Mg^2+^-nucleotide-regulated metal ion current [7]) is measurable in virtually all cell types examined [4,16], native currents of the more limitedly expressed TRPM6 have not yet been reported. TRPM7 channels are indispensable for Mg^2+^ homeostasis at both cellular and whole organism levels [17,18]. In contrast, TRPM6 is believed to be responsible for systemic Mg^2+^ regulation by mediating Mg^2+^ (re) absorption, as a mutation in the TRPM6 gene leads to an autosomal recessive form of familial hypomagnesemia with secondary hypocalcemia [19,20]. Consistent with its physiological function, TRPM6 expression appears to be relatively tissue-restricted, predominantly in the absorptive epithelia in the colon and kidney [6,19,20]. However, it is unknown whether TRPM6 is aberrantly expressed in other tissues or cells, possibly in a pathophysiological context.

Neuroblastoma, arising in tissues of the sympathetic nervous system such as sympathetic ganglia and the adrenal medulla, is the most common extracranial solid tumor of childhood and accounts for around 15% of all pediatric cancer deaths [21-23]. A factor that is strongly associated with advanced high-risk neuroblastoma and predicts poor outcome is amplification and concomitant high expression of MYCN [24,25], an oncogene encoding N-Myc, a nuclear phosphoprotein in the Myc family of helix-loop-helix transcription factors [26]. Enhanced expression of the oncoprotein N-Myc regulates a large number of genes, disrupting the cell cycle exit and terminal differentiation of neuroblasts and hence promoting neuroblastoma pathogenesis [27,28]. Given the role of Mg^2+^ in cell proliferation, the present study carefully examined two Mg^2+^-transporting channel kinases, TRPM7 and TRPM6, in neuroblastoma cells. Our results reveal that TRPM7 is essential for neuroblastoma proliferation and the additional expression of TRPM6 modulates the phenotype of native MagNuM currents, favoring divalent cation transport mediated by TRPM6 and TRPM7 and promoting neuroblastoma cell proliferation.

## RESULTS

### N-Myc regulates expression of TRPM6 and TRPM7 in human SHEP-21N neuroblastoma

**Figure 1:**
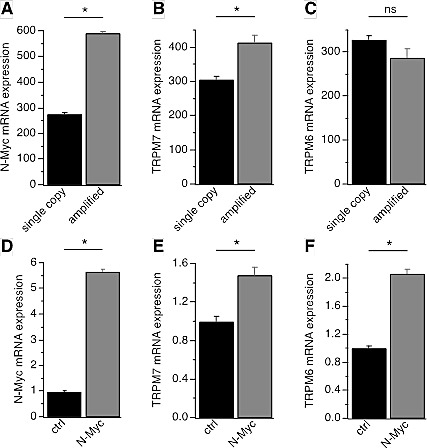
TRPM6 and TRPM7 correlation with MYCN in neuroblastoma A-C, N-Myc, TRPM7 and TRPM6 mRNA expression correlation with MYCN amplification in the Kocak-649 cohort. Microarray analysis of N-Myc, TRPM7 and TRPM6 mRNA expression in Kocak-649 (GSE45547), the largest neuroblastoma cohort in the public domain. The graphs present N-Myc (A), TRPM7 (B) and TRPM6 (C) expression in tumors without (n=550) and with (n=93) MYCN amplification. Y-axes represent sample ranks in a non-parametric Kruskal-Wallis t test; actual mean ± s.e.m. expression values were: 17,839 ± 1,137 (MYCN), 104.9 ± 3.7 (TRPM6), 807.0 ± 321.1 (TRPM7). Both N-Myc and TRPM7 expression are significantly higher in tumors with MYCN amplification (*p*=1.5 • 10^−51^ and *p*=2.3 • 10^−7^ respectively; Kruskal-Wallis t test). D-F, qRT-PCR analysis of N-Myc (D), TRPM7 (E) and TRPM6 (F) expression levels in the SHEP-21N cell line where MYCN transgene is controlled by tetracycline, i.e., N-Myc expression is repressed in the presence of tetracycline (control), but removal of tetracycline induces N-Myc expression (N-Myc). The graphs show normalized N-Myc, TRPM7 and TRPM6 expression in SHEP-21N cells with (N-Myc, n = 9) or without (control, n = 9) N-Myc expression. *, *p*<0.01.

We set out to examine the expression level of the Mg^2+^-influx channels TRPM6 and TRPM7 in the largest publicly available neuroblastoma expression profiling dataset, the Kocak-649 cohort [29]. On the basis of their MYCN gene copy number status, these tumor samples can be divided into MYCN-amplified (n=93) or non-MYCN-amplified (n=550) tumors. We first examined the expression level of N-Myc in these two groups and found that the expression level is, as expected, significantly higher in the MYCN-amplified samples (*p* = 1.5 × 10^−51^, Fig. 1A). Interestingly, TRPM7 mRNA expression was found in all tumor samples, and was significantly correlated with both MYCN amplification (*p* = 2.3 × 10^−7^, Fig. 1B) and mRNA expression (*p* = 3.5 × 10^−3^ in a 2logPearson test). No significant correlations were found for TRPM6 (Fig. 1C), most likely due to the small numbers of tumors with significant TRPM6 expression (average TRPM6 expression was 8-times lower than that of TRPM7). In order to investigate the role of N-Myc in TRPM7/TRPM6 regulation, we chose the SHEP-21N cell line, a clone derived from the SHEP-2 neuroblastoma, in which N-Myc is constitutively expressed but can be experimentally repressed [30]. These cells contain a MYCN trans-gene under the control of a tetracycline-responsive repressor element, so that tetracycline exposure turns off N-Myc expression. Quantitative RT-PCR (qRT-PCR) analysis showed that SHEP-21N cells without N-Myc expression had basal expression of TRPM7 and TRPM6, which was considerably enhanced by N-Myc up-regulation (Figs. 1D-F). Induction of TRPM6 and TRPM7 expression by N-Myc was significant for both channel kinases, but higher for TRPM6 (2.1-fold for TRPM6 and 1.5-fold for TRPM7). This suggests that N-Myc up-regulates both TRPM genes and concomitantly increases the ratio of TRPM6 over TRPM7.

### Endogenous MagNuM currents in SHEP-21N are mediated by TRPM6 and TRPM7

**Figure 2:**
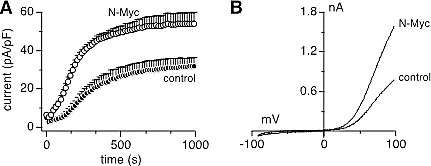
Endogenous MagNuM currents are increased by N-Myc upregulation Currents were measured in divalent-free internal solution with 5 mM EGTA and 5 mM EDTA. A, whole-cell current development in SHEP-21N cells expressing (N-Myc, n = 11) or not (control, n = 10). B, corresponding representative ramp currents (I-V curves) extracted at 1,000 s from both groups.

**Figure 3:**
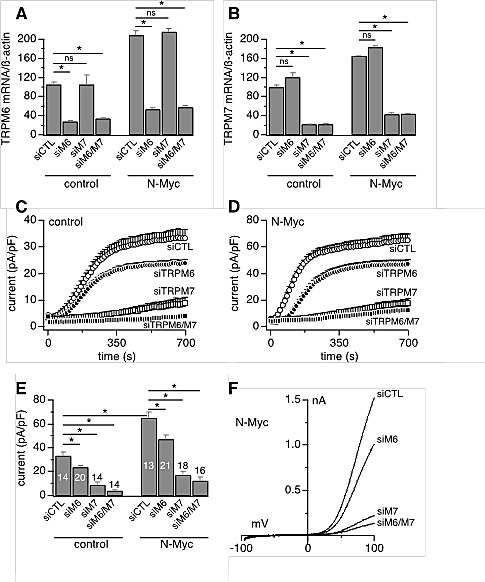
Endogenous MagNuM currents are mediated by TRPM7 and TRPM6 Currents were measured in divalent-free internal solution with 5 mM EGTA and 5 mM EDTA. A-B, siRNAs efficacy measured by qRT-PCR analysis of TRPM6 (A) and TRPM7 (B) expression levels in the SHEP-21N cell line under both control and N-Myc upregulation conditions (n = 9). C, MagNuM currents in control SHEP-21N cells treated with negative non-silencing (siCTL) or specific siRNA sequences against TRPM7, TRPM6, TRPM7&TRPM6. D, current measurement in N-Myc-expressing SHEP-21N cells treated with negative (siCTL) or specific siRNA sequences against TRPM7, TRPM6, TRPM7&TRPM6. E, statistical summary of current amplitudes at 700 s as in c-d (*, *p*<0.01). The number of patched cells is indicated in graph. F, typical current traces evoked by voltage ramps (I-V curves) in N-Myc-expressing SHEP-21N cells as in D.

Heterologous expression of heteromeric TRPM7/TRPM6 produces an outwardly rectifying current whose current-voltage relationship is indistinguishable from homomeric TRPM7 or TRPM6 currents [9,14]. Patch-clamp recordings in SHEP-21N cells also revealed such currents (Figs. 2A, B). Importantly, SHEP-21N cells exhibited significantly larger current amplitudes when they expressed N-Myc (Figs. 2A, B), consistent with the observed N-Myc-induced increases in TRPM7 and TRPM6 mRNA levels (Figs. 1E, F). Employing specific small interfering RNA (siRNA) against TRPM7 and TRPM6 (Figs. 3A, B), we found that MagNuM currents were significantly suppressed upon TRPM6 knockdown, and both knockdown of TRPM7 or TRPM7/TRPM6 combined nearly abolished any endogenous MagNuM currents (Figs. 3C-F). These results demonstrate that N-Myc expression produces significantly larger overall MagNuM currents and suggest that, independent of N-Myc levels, endogenous MagNuM currents in SHEP-21N cells are largely composed of TRPM7 with a lesser, yet significant, contribution of TRPM6.

### N-Myc expression shapes the phenotype of native TRPM7/TRPM6 currents

**Figure 4:**
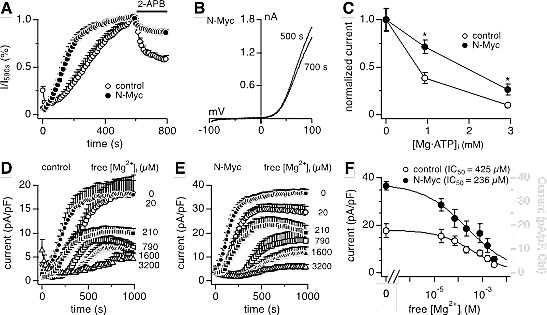
N-Myc expression shapes the phenotype of native TRPM7/TRPM6 currents Whole-cell MagNuM currents were measured in SHEP-21N cells treated with tetracycline (control) or not (N-Myc) and peak outward currents at +80 mV were analyzed. A, inhibition of MagNuM current by 200 μM 2-APB (control, n = 10; N-Myc, n = 7), normalized to current amplitude just prior to 2-APB application. B, representative currents evoked by voltage ramps (I-V curves) were derived from N-Myc-expressing cells before (500 s) and after (700 s) 2-APB application. C, ATP sensitivity of currents (n = 7-13 cells for each point; *, *p*<0.01). Mg·ATP concentrations were fixed at 1 or 3 mM with an internal free Mg^2+^ concentration of 264 μM. Currents were normalized against the control condition containing no Mg·ATP internally. D, E, current measurements in control and N-Myc-expressing cells perfused with intracellular solutions of defined free Mg^2+^ concentrations as labeled in the graph (control, n = 5-6 for each Mg^2+^ concentration; N-Myc, n = 5-7 for each Mg^2+^ concentration). Free Mg^2+^ concentration in internal solution was clamped to the indicated levels with 10 mM EGTA. F, peak current amplitudes derived from D and E as a function of free Mg^2+^ concentration. Dose-response fit rendered IC_50_ values of 425 μM and 236 μM for control and N-Myc-expressing cells, respectively.

Heteromeric TRPM7/TRPM6 channels behave differently from both homomeric TRPM7 and homomeric TRPM6. In heterologous expression systems, 2-APB suppresses TRPM7 currents, potentiates TRPM6 currents, and leaves heteromeric TRPM7/TRPM6 largely unaffected, indicating that incorporation of TRPM6 renders the TRPM7/TRPM6 heteromer less sensitive to 2-APB [9,14]. Indeed, N-Myc induction attenuated the inhibitory effects of 2-APB on the MagNuM currents in SHEP-21N cells (Figs. 4A, B). Since homomeric TRPM6 channels are strongly suppressed by free intracellular Mg^2+^, heteromeric TRPM7/TRPM6 channels exhibit increased sensitivity of Mg^2+^-mediated suppression relative to TRPM7. This has been shown to be due to cross-phosphorylation of TRPM7 by the TRPM6 kinase domain [14]. Consistent with this mechanism, the dose-response curves for intracellular free Mg^2+^ in SHEP-21N cells revealed that N-Myc expression and the resulting up-regulation of TRPM6 considerably enhanced the sensitivity of native currents to Mg^2+^, as indicated by a decrease in IC_50_ from 425 μM to 236 μM (Figs. 4D-F). Finally, and physiologically most relevant, intracellular Mg·ATP strongly suppresses heterologously expressed TRPM7 channels [7], but has no inhibitory effect on either TRPM6 or TRPM7/TRPM6 currents [14]. Accordingly, N-Myc expression in SHEP-21N cells significantly reduced Mg·ATP-mediated suppression of endogenous MagNuM currents (Fig. 4C). Thus N-Myc expression caused changes in the behavior of native MagNuM currents in SHEP-21N cells with respect to 2-APB, intracellular free Mg^2+^ and Mg·ATP that resemble the channel phenotype of overexpressed TRPM7/TRPM6 heteromers [14] and consequently would favor enhanced constitutive activity of these channels at rest.

### Suppression of TRPM7/TRPM6 inhibits proliferation of neuroblastoma

**Figure 5:**
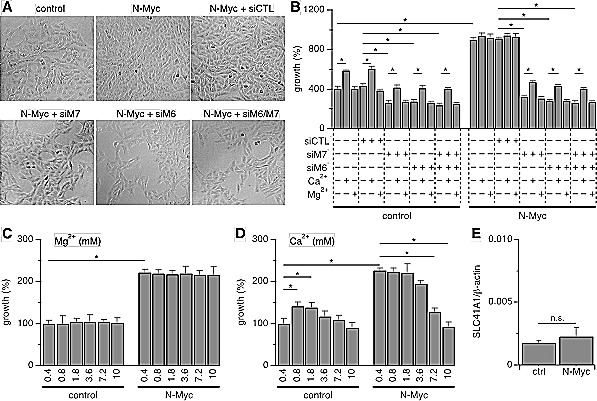
siRNA, Ca^2+^ and Mg^2+^ supplementation and TRPM7/M6-dependent cell proliferation A, representative images of SHEP-21N cells treated with siRNA as indicated. Tetracycline was added (control) or not (N-Myc) to repress or induce N-Myc expression, respectively. siCTL = negative control siRNA; siM6 = siRNA against TRPM6; siM7 = siRNA against TRPM7. B, statistical analysis of cell growth in SHEP-21N cells treated with the indicated siRNA (n = 6; *, *p*<0.01). Cells were transfected with siRNA, seeded, treated or not treated with Mg^2+^ or Ca^2+^ on day 1, and analyzed on day 3 after seeding. C-D, analysis of cell growth in SHEP-21N cells treated for 2 days with the indicated Mg^2+^ (C) or Ca^2+^ (D) concentrations (n = 6). E, qRT-PCR analysis of SLC41A1 transcripts in both control and N-Myc-expressing SHEP21N cells (n = 6; n.s., not significant).

We performed knockdown experiments using siRNA as described above to investigate the relative role of both TRPM6 and TRPM7 channels in SHEP-21N cell proliferation. N-Myc induction following tetracycline withdrawal promoted cell proliferation compared to tetracycline-treated SHEP-21N cells without N-Myc expression (Figs. 5A, B). In either group, the additional siRNA-mediated knockdown of TRPM7, TRPM6 or TRPM7/TRPM6 expression markedly inhibited SHEP-21N cell proliferation (Figs. 5A, B). Notably, knockdown of TRPM6 alone strongly inhibited cell proliferation, an effect that was comparable to that seen with TRPM7 suppression (Figs. 5A, B). Given that TRPM6 knockdown only causes moderate reduction in total MagNuM currents recorded under divalent-free intracellular solutions (Figs. 3C, D), this indicates that TRPM6 may play a vital role in promoting cell proliferation under physiological conditions by enhancing the constitutive activity of MagNuM currents.

Since TRPM6 and TRPM7 are permeable to both Ca^2+^ and Mg^2+^ and both divalent cations can regulate cell proliferation, we assessed the role of Ca^2+^ and Mg^2+^ in SHEP-21N proliferation assays. We first established a dose-response relationship of Ca^2+^ and Mg^2+^ by incubating SHEP-21N cells in media with Ca^2+^ and Mg^2+^ concentrations adjusted in the range of 0.4 mM to 10 mM (while the congeneric divalent remained at 0.4 mM). Increasing Mg^2+^ up to 10 mM had no effect on SHEP-21N cell proliferation regardless of N-Myc expression (Fig. 5C). Increasing Ca^2+^ levels up to 1.8 mM had no significant effect on N-Myc-expressing cells, but yielded a slight increase in cell numbers in cells without N-Myc expression (Fig. 5D). At higher concentrations of Ca^2+^, cell proliferation started to decline in both cell populations. For this reason, we assessed possible compensatory effects of divalent supplementation on cell proliferation in cells in which TRPM6, TRPM7, or both, were knocked down with siRNA using concentrations of 10 mM Mg^2+^ and 1.8 mM Ca^2+^ (Fig. 5B). As illustrated in Fig. 5B, 10 mM Mg^2+^ had no effect on cell proliferation, indicating the low abundance or absence of alternative Mg^2+^ uptake mechanisms in these cells. Indeed, qRT-PCR analysis of SLC41A1, a Mg^2+^ transporter that has been found to rescue TRPM7-deficient DT40 cells from growth arrest [17,31], revealed that its mRNA levels were 123/24-fold less expressed than those of TRPM7/TRPM6, respectively (Figs. 5E and 1E, F). Importantly, *SLC41A1* mRNA levels did not change significantly upon N-Myc induction. Interestingly, Ca^2+^ supplementation to 1.8 mM partially rescued the growth-arrest phenotype by causing a slight increase in cell numbers under all experimental conditions, except in N-Myc-expressing SHEP-21N cells in which TRPM6 and TRPM7 were left uninhibited and which were already strongly proliferating. However, neither cation was able to decisively rescue the growth suppression imposed by the knockdown of either channel. Thus, TRPM7, particularly in combination with TRPM6, appears to represent the major Ca^2+^ and/or Mg^2+^ accumulation mechanisms in SHEP-21N cells.

## DISCUSSION

While TRPM6 was initially discovered in the absorptive epithelia in the kidney and intestine [5,6], the present study now demonstrates that the protein is also expressed in SHEP-21N neuroblastoma. TRPM6 expression in SHEP-2 and SHEP-21N is not serendipitous, as data mining of Kocak-649 and other public neuroblastoma cohort expression profiles showed very similar expression levels and patterns: TRPM7 mRNA expression is ubiquitous and TRPM6 mRNA is much lower, and present in only ~10% of samples (results not shown). Remarkably, SHEP-21N cells expressed TRPM6 at a sufficiently high level that endogenous TRPM6-dependent currents could be measured for the first time. The central observation made in the present study was that the enhanced proliferation phenotype mediated by N-Myc expression (Figs. 1 and 5A, B) was paralleled by increased TRPM6 and TRPM7 expression levels (Figs. 1E, F), as well as by enlarged endogenous MagNuM currents (Figs. 2A, B). The phenotypic characteristics of the resulting currents are consistent with heteromeric TRPM6/TRPM7 channels (Fig. 4). Molecular suppression of either channel through siRNA (Figs. 3C-F and 5A, B) completely suppressed both the N-Myc-enhanced MagNuM currents and the growth phenotype (Figs. 3 and 4).

Even though both TRPM6 and TRPM7 also possess kinase activity in addition to their channel function, TRPM6 has no known phosphorylation substrates other than TRPM7 [32] and the currently known TRPM7 substrates, annexin A1 and myosin IIA heavy chain [33,34], are not likely to determine cell proliferation. This indicates that enhanced ion transport activities are in part responsible for the growth phenotype observed in N-Myc-expressing SHEP-21N cells. Our results are therefore consistent with the hypothesis that N-Myc enhances TRPM6 expression, which in turn promotes Ca^2+^ and Mg^2+^ transport across the plasma membrane by enhancing the overall activity of TRPM6/TRPM7 heteromeric channel assemblies, and thereby promotes cell proliferation.

The expression patterns of TRPM6 and TRPM7 in MYCN-amplified neuroblastoma cell lines and SHEP-21N cells suggests that their expression might not necessarily relate to N-Myc expression, as some MYCN-amplified cell lines had low TRPM6 expression levels (data not shown). Nevertheless, in SHEP-21N cells, N-Myc is able to elevate basal TRPM6/7 expression upon induction. In addition, in the largest publicly available neuroblastoma expression profiling dataset, the Kocak-649 cohort [29], TRPM7 mRNA expression is found in all tumor samples, and is significantly correlated to N-Myc amplification. No significant correlations were found for TRPM6, most likely due to the small numbers of tumors with significant TRPM6 expression. Future investigations might reveal oncogene-dependent increases of TRPM6 expression in other cells that already express some basal levels of TRPM6.

We previously demonstrated in heterologous expression systems that TRPM7/TRPM6 channel heteromers, when co-expressed at a stoichiometric ratio of ~1:1, exhibit unique phenotypes that are characterized by insensitivity to 2-APB, lack of modulation by Mg·ATP and increased sensitivity to Mg^2+^ inhibition compared with homomeric TRPM7 [14]. The insensitivity of heteromeric TRPM7/TRPM6 channels to cytosolic ATP leaves the channel function largely unaffected by cellular energy status and augmented Mg^2+^ inhibition indicates an enhanced negative feedback loop, a common regulatory mechanism for many ion channels, such as CRAC and TRPV5/TRPV6 [35,36]. These distinctive features of heteromeric TRPM7/TRPM6 channels are primarily controlled by cross-phosphorylation of TRPM7 by the TRPM6 kinase domain [32] and will determine the functional activity of the channel complex as a Ca^2+^ and Mg^2+^ influx conduit under physiological circumstances. Consistent with the findings in TRPM7/TRPM6 overexpression systems, the native currents in SHEP-21N cells expressing N-Myc were less inhibited by 2-APB, less suppressed by Mg·ATP, and more sensitive to Mg^2+^ inhibition compared to the currents in cells without N-Myc expression (Fig. 4). Thus, N-Myc shaped the MagNuM phenotype towards that of heteromeric TRPM7/TRPM6 by increasing the relative amount of TRPM6.

It is noteworthy that the extent to which the MagNuM phenotype was altered in native SHEP-21N cells is slightly less pronounced than that previously observed in a TRPM7/TRPM6 overexpression system, where the ratio of TRPM6/TRPM7 was ~1 [14,32], suggesting that the ratio of TRPM6 over TRPM7 within channel complexes in SHEP-21N cells is lower than 1. This would be consistent with the observed mRNA levels (Figs. 1E, F) and also be consistent with the observation that TRPM6 suppression downregulated the MagNuM currents by 29%, whereas knockdown of TRPM7 reduced current amplitudes by 74% (Fig. 4). Nevertheless, the impact of TRPM6 knockdown on cell proliferation was as severe as that of TRPM7, suggesting that relatively small increases in TRPM6 expression can significantly alter the MagNuM current behavior under physiological conditions. Since TRPM6/TRPM7 heteromers are less sensitive to Mg·ATP and slightly more sensitive to Mg^2+^ inhibition (Fig. 4; [14]), TRPM6-containing heteromeric channels in SHEP-21N would likely be less suppressed under physiological conditions, as the most dominant physiological regulator of MagNuM is Mg·ATP [7]. As a result, these heteromeric channels would be more effective in supporting proliferation than TRPM7 homomers.

TRPM7 and TRPM6 conduct divalent cations with preferential transport of Mg^2+^ over Ca^2+^. Consistent with this notion is that TRPM6 plays a well-documented role in Mg^2+^ absorption/reabsorption in epithelia of colon and kidney [6,19,20] and TRPM7 has been found to be critical for cellular Mg^2+^ homeostasis [17,18] and a critical factor for cell proliferation [13,37,38]. The presence and regulation of TRPM6 in SHEP-21N cells thus would suggest that this channel may also serve a role in the pathological context of neuroblastoma by regulating cellular Mg^2+^ levels in conjunction with TRPM7. However, the role of Ca^2+^ transport through TRPM6 and/or TRPM7 may not be insignificant, particularly in cells in which Ca^2+^ is an important regulator of cell proliferation. Thus, it has been suggested that Ca^2+^ influx through TRPM7 may be critical for fibroblast proliferation [39] and a recent study on prostate cancer cells proposed that enhanced Ca^2+^ influx through TRPM7 determined their proliferation rate [40].

Our Ca^2+^ and Mg^2+^ supplementation experiments revealed that increasing extracellular Mg^2+^ from 0.4 to 10 mM had no effect on proliferation regardless of N-Myc expression in SHEP-21N cells (Fig. 5), whereas Ca^2+^ had a slight facilitatory effect up to 1.8 mM. Higher concentrations of Ca^2+^ reduced cell proliferation. Extracellular Mg^2+^ can be taken up by mammalian cells through Mg^2+^-permeable channels, including TRPM7 and TRPM6 [5], and/or Mg^2+^ transporter, such as SLC41A1 [41]. Genetic ablation of TRPM7 in DT40 cells induces growth arrest, and supplementation with high extracellular Mg^2+^ (10 mM), but not Ca^2+^, rescues the growth arrest [42], a compensatory effect partly mediated by the Mg^2+^ transporter SLC41A1 that is natively expressed in DT40 cells [31]. However, Mg^2+^ supplementation was completely ineffective in overcoming the growth arrest of SHEP-21N cells in which either TRPM6, TRPM7, or both were knocked down with siRNA (Fig. 5B), suggesting that TRPM6/TRPM7 channels, rather than SLC41A1, constitute the major Mg^2+^ uptake mechanism in these cells. An alternative explanation could be that Mg^2+^ may not be the limiting factor for cell proliferation in these cells and the more relevant cation might be Ca^2+^. Indeed, supplementation of Ca^2+^ (1.8 mM) in the extracellular solution slightly enhanced proliferation, although not in N-Myc-expressing SHEP-21N cells. The reason for this could be that these SHEP-21N cells already proliferated at maximal rates. It remains to be determined whether the facilitatory effect of Ca^2+^ is mediated by Ca^2+^ influx through TRPM6/TRPM7 channels or represents an alternative Ca^2+^ influx pathway that offsets a Mg^2+^-dependent growth impairment.

The data presented in this study suggest that TRPM7 channels have a significant impact on cell proliferation of neuroblastoma, regardless of whether or not they express N-Myc, whereas additional TRPM6 expression further synergizes with TRPM7 in augmenting the proliferative activity. The relevance of TRPM7 for cell proliferation is also evident in other cancers, as genetic suppression of TRPM7 can inhibit the proliferation of human head and neck squamous carcinoma (HNSCC) [37] and ascending aortic vascular smooth muscle cells (VSMC) [38]. Pharmacological inhibition of TRPM7 by the channel blocker waixenicin A has also been shown to inhibit cell proliferation of rat basophilic and human Jurkat T-cell leukemia cells [13]. This makes Ca^2+^/Mg^2+^-transporting channels attractive candidates as pharmacological targets for potential therapeutic drugs. Collectively, genetic or pharmacological suppression of TRPM7/TRPM6 channels inhibits cell proliferation, underscoring the importance of these channels in promoting proliferation and pointing towards a potential therapeutic avenue for the treatment of neuroblastoma.

## MATERIALS AND METHODS

### Cell culture and microarray analysis

Neuroblastoma cell line and culture conditions were as in [28,43]. N-Myc expression in SHEP-21N [28,30] was inhibited by the addition of tetracycline (100 ng/ml) and incubation for at least 48 hours. The Agilent-020382 Human Custom Microarray 44k data for Kocak-649 was described in [29]. Expression data for this dataset were retrieved from the public Gene Expression Omnibus (GEO) dataset on the NCBI website (GEO ID: GSE45547). The TranscriptView genomic analysis and visualization tool was used to confirm the anti-sense position in a (posterior) exon of the probe-sets. The Agilent probe-sets MYCN 24_P94402 (MYCN), 24_P410463 (TRPM6), and 23_P88470 (TRPM7), that fulfilled these criteria, and in addition showed the highest sensitivity for detection of the target gene, were selected. Alternative correct probe-sets did not yield conflicting results. All analyses were performed using R2 (http://r2.amc.nl).

### Electrophysiology and solutions

Whole-cell patch-clamp experiments were performed at room temperature (20-25 °C). High-resolution whole-cell currents were recorded by EPC-9 (HEKA, Bellmore, NY) and Patchmaster v2.4 (HEKA). Unless otherwise stated, all voltages were corrected for a liquid junction potential of 10 mV. Patch pipettes pulled from borosilicate glass had resistances of 2.5-3.5 MΩ when filled with internal solutions. Cells were held at 0 mV holding potential, with voltage ramps of 50 ms duration spanning the voltage range of -100 mV to +100 mV delivered at a rate of 0.5 Hz following the establishment of whole-cell configuration. The currents were filtered at 2.9 kHz and digitized at 10 kHz. SHEP-21N cells plated on coverslips were bathed in Mg^2+^-free external solution containing (in mM) 140 NaCl, 2.8 KCl, 1 CaCl_2_, 10 HEPES, 11 Glucose, pH 7.4 adjusted with NaOH. The divalent-free internal recording solution was composed of (in mM): 140 Cs-glutamate, 8 NaCl, 5 Cs-EDTA, 5 Cs-EGTA, 10 Cs-HEPES, pH 7.2 adjusted with CsOH. When needed, internal free Mg^2+^ was clamped by 10 mM EGTA to various levels, as calculated by WebMaxC (http://maxchelator.stanford.edu).

### RNA extraction and quantitative real-time PCR (qRT-PCR)

Quantitative real-time PCR (qRT-PCR) was used to examine the mRNA expression levels of TRPM6 and TRPM7 using β-actin for normalization. Total RNA (1 μg) was extracted from SHEP-21N cells treated with or without tetracycline using the RNeasy Mini kit (Qiagen, Valencia, CA). Conversion of mRNA to cDNA was achieved using random priming by ABI's High Capacity cDNA RT Kit with RNase Inhibitor (Applied Biosystems, Life Technologies, Foster City, CA). The qRT-PCR was performed using the ABI HT7900 FAST Real-Time PCR System (Applied Biosystems) and ABI POWER SYBRGreen (Applied Biosystems) according to the manufacturers' protocols. Gene-specific primer pairs of human MYCN (QT00201404) human TRPM6 (QT00043456), TRPM7 (QT00082425) and β-actin (QT01680476) were purchased from Qiagen.

### RNA interference and cell proliferation assay

The small interfering RNA (siRNA) duplexes specific against human TRPM6 (4392420) and TRPM7 (4390824), and a non-silencing RNA sequence (4390843) as a negative control were transiently transfected with RNAiMAX (Invitrogen) according to the manufacturer's protocols. siRNA transfection and cell seeding were carried out simultaneously, according to the reverse transfection protocol of RNAiMAX. Briefly, siRNA at a final concentration of 20 nM, diluted in Opti-MEM (Invitrogen), was mixed with RNAiMAX in 6-well plates, followed by seeding of SHEP-21N cells at 5×10^4^ cells/well in 6-well plates,. On day 2 after siRNA transfection, micrographs of SHEP-21N cells were taken at ×10 magnification. Currents were measured and cell numbers counted on day 3 after transfection. To count the cell number, SHEP-21N cells were trypsinized to single cells and stained with trypan blue, followed by cell counting with a hemocytometer. Each well was counted four times. The experiments were repeated as indicated in the figure legends.

### Statistical analysis

Currents were analyzed with FitMaster v2.11 (HEKA) and Igor Pro (Wavemetrics, Portland, OR). Peak outward currents at +80 mV were extracted for statistical analysis. Current amplitudes were normalized to cell size upon break-in as current density (pA/pF). Dose-response curves were calculated using the function *f*(x) = (Y_max_*(1/(1+(IC_50_/x)^n^))), where Y_max_ is the maximal normalized current, IC_50_ is the concentration at which inhibition is half maximal, x is the concentration, and n is the Hill coefficient. All data are given as mean ± standard error of mean (s.e.m.). Appropriate ANOVA, Student's, or Kruskal-Wallis *t* tests, and 2logPearson correlation were performed to assess statistical significance. *P* values <0.05 were considered as statistically significant.
